# Body Weight Status and Dietary Intakes of Urban Malay Primary School Children: Evidence from the Family Diet Study

**DOI:** 10.3390/children4010005

**Published:** 2017-01-20

**Authors:** Wai Yew Yang, Tracy Burrows, Lesley MacDonald-Wicks, Lauren T. Williams, Clare E. Collins, Winnie Siew Swee Chee, Kim Colyvas

**Affiliations:** 1 School of Health Sciences, Faculty of Health and Medicine, and Priority Research Centre in Physical Activity and Nutrition, The University of Newcastle, Callaghan, NSW 2308, Australia; tracy.burrows@newcastle.edu.au (T.B.); lesley.wicks@newcastle.edu.au (L.M.-W.); lauren.williams@griffith.edu.au (L.T.W.); clare.collins@newcastle.edu.au (C.E.C.); 2 Division of Nutrition and Dietetics, School of Health Sciences, Faculty of Medicine and Health, International Medical University, Kuala Lumpur 57000, Malaysia; winnie_chee@imu.edu.my; 3 Menzies Health Institute of Queensland, Griffith University, Gold Coast Campus, QLD 4222, Australia; 4 School of Mathematical and Physical Sciences, Faculty of Science and Information Technology, The University of Newcastle, Callaghan, NSW 2308, Australia; kim.colyvas@newcastle.edu.au

**Keywords:** child, diet, nutrition, body weight, energy misreporting, developing country

## Abstract

Malaysia is experiencing a rise in the prevalence of childhood obesity. Evidence for the relationship between dietary intake and body weight among Malaysian children is limited, with the impact of energy intake misreporting rarely being considered. This paper describes the dietary intakes of urban Malay children in comparison to national recommendations and by weight status. This cross-sectional Family Diet Study (*n* = 236) was conducted in five national primary schools in Malaysia (August 2013–October 2014). Data on socio-demographics, anthropometrics, 24-h dietary recalls, and food habits were collected from Malay families, consisting of a child aged 8 to 12 years and their main caregiver(s). Multivariable analyses were used to assess dietary intake-body weight relationships. The plausibility of energy intake was determined using the Black and Cole method. Approximately three in 10 Malay children were found to be overweight or obese. The majority reported dietary intakes less than national recommendations. Children with obesity had the lowest energy intakes relative to body weight (kcal/kg) compared to children in other weight categories (F = 36.21, *p* < 0.001). A positive moderate correlation between energy intake and weight status was identified (*r* = 0.53, *p* < 0.001) after excluding energy intake mis-reporters (*n* = 95), highlighting the need for the validation of dietary assessment in obesity-related dietary research in Malaysia.

## 1. Introduction

Childhood obesity is a global health challenge of the 21st century, especially in developing countries [1]. Although the overall prevalence is higher in developed countries, there have been large increases in childhood obesity in developing countries [2]. A systematic review of childhood overweightness in developing countries reported the highest prevalence rates in the Middle East (89.6%) and Eastern Europe (48.4%) [3]. Within Asia, there was a relatively low prevalence of childhood obesity prior to the millennium [4,5]; however the problem is likely to be understated, given the large population density and the rate of economic development in this region since [1,6,7]. Malaysia is a developing Asian country that is experiencing a rapidly rising prevalence of childhood obesity, consistent with international trends [1]. A nationwide study published in 2013 found that approximately 34.5% of urban primary school-aged children were either overweight or obese [8], similar to the data reported from developed countries [1]. Although no significant ethnic differences have been reported, the magnitude of child obesity may be greater for children of Malay ethnicity compared to other ethnicities, based on their proportion within the Malaysian population (67.4% Malay versus (vs.) Chinese 24.6%; Indian 7.6%; and others 0.7%) [9]. The adverse consequences of childhood obesity are well documented [10] and include a life expectancy estimated to be seven years shorter [11]. A recent review on childhood obesity and obstructive sleep apnea reported that the prevalence of obstructive sleep apnea amongst overweight and obese children could be as high as 60% [12]. Epidemiological data showed that the prevalence of type 2 diabetes is increasing globally, with children from specific ethnic groups at increased risk compared to white children [13,14,15]. Alongside rising childhood obesity and type 2 diabetes, the prevalence of metabolic syndrome in children is increasing in most developing countries [15].

The literature to date on childhood obesity suggests a multi-factorial etiology [15,16]. Although the precise cause of childhood obesity in developing countries has not been elucidated, it is thought to be predominantly driven by environmental factors rather than genetics [17,18,19], given the rapid prevalence increase in a relatively stable population within a short time frame [16]. A review on worldwide trends of childhood obesity suggested that several characteristics emerging within developing countries, including urbanization, Westernized lifestyle, and rapid socio-economic development, were associated with an increased risk of overweightness and obesity in children [6]. Diet is an important contributor to energy imbalance and specific eating patterns, including the increased consumption of sweetened beverages and candies (highly refined carbohydrates) and processed meats (animal protein, oils, and fats) have been shown to be associated with overweight status amongst children in the United States [20]. With economic globalization, there is a significantly increased availability of Western-style energy-dense and nutrient-poor processed foods in developing countries in Asia [16,21]. Over the past two decades, Malaysia has undergone rapid nutrition and lifestyle transitions, characterised by a shift away from the high energy activities of daily living to a sedentary lifestyle and from a traditional grain-based diet (high in complex carbohydrates and fiber) to one high in animal products, oils, and fats, and lower in fiber [17]. These obesogenic factors in the environment influence food behavior and increase the incidence of childhood obesity in developing countries [10,15].

To date, little has been reported on the relationship between dietary intake and obesity within developing Asian countries [17]. The literature that does exist is equivocal with some studies demonstrating a relationship between diet and body weight status, specifically that higher intakes of fruit and vegetables are associated with lower weight status [22,23]. Others have found no relationship between daily breakfast intake and the risk of overweightness/obesity [24,25]. A systematic review found the associations between diet and childhood obesity to be inconclusive, primarily due to a limited number of studies [7] and methodological issues regarding the use of non-validated dietary assessment tools [26].

Malaysia had no national nutrition and dietary intake data for children under 12 years of age until a 2013 publication [8]. The current literature on dietary intake of Malaysian children is limited, with published studies reporting on a selective dietary profile either of nutrient intakes, food habits, or individual dietary practices [8,27,28,29]. Accurately assessing the dietary intake of children is a difficult task [30] and the misreporting of energy intake among Malay children has been shown to be common [31]. These factors could confound the link between diet and weight status. Therefore, the aim of this paper is to describe the dietary intakes of primary school-aged Malay children with plausible energy intakes, in comparison to national recommendations and by body weight status.

## 2. Methods

### 2.1. Study Design and Participants

The Family Diet Study used a cross-sectional design and was conducted from August 2013–October 2014 in the urbanized areas of Peninsular Malaysia, Kuala Lumpur (100% urbanisation) and Selangor (91.4% urbanisation)) [9]. The full study methodology is detailed elsewhere [32]. Briefly, the approval was obtained from the ethics committees of the University of Newcastle, Australia (H-2013-0065) and the International Medical University, Malaysia (IMU 275/2013). In Malaysia, compulsory schooling commences at age seven and the proportion of children enrolled in primary school was 94% [33]. Primary school thus provided an excellent setting for the recruitment of children in the targeted age range of 8 to 12 years.

The Malaysian education ministry and departments gave permission to contact nominated schools. Multi-stage sampling was used for recruitment, including convenience, simple random, and cluster sampling. The location was chosen based on convenience and includes two different states located in the central of Peninsular Malaysia; Kuala Lumpur (1.67 million populations and 100% urbanisation) and Selangor (5.46 million populations and 91.4% urbanisation). The selection of eight national primary schools within the identified zone was based on a simple random sampling method, while cluster sampling was implemented to select a minimum of three primary schools from each zone. Using the prevalence of breakfast skipping (14%) from the pilot study data and EPI-info™ (Version 5.0), the study sample size required was estimated to be 220 participants (5% margin of error and 95% confidence level).

Interested families provided informed written consent with child assent and were screened for eligibility based on study inclusion/exclusion criteria. The study participants included Malay families with one or two main caregiver(s) living full time with a child between 8 and 12 years old (Primary 3, 4 and 5). Exclusion criteria included children with known medical conditions that could influence body weight, metabolic rate, or appetite, including asthma, type 1 diabetes, or the use of medications associated with weight change such as oral steroids. Data on the children is reported in this paper. Participants were given book vouchers as tokens of appreciation following data collection and written feedback was provided to the families on their individualised anthropometry, dietary intake, and food habit results.

### 2.2. Measurements

#### 2.2.1. Socio-Demographic

Each participating family (either one or both parents and their child) completed a written survey that collected data on socio-demographics, including family size, the family’s monthly total income, weekly pocket money provided to the participating child, and parental employment status and highest education level.

#### 2.2.2. Anthropometry

The children were weighed in light clothing on portable scales (TANITA, Tokyo, Japan). Height was measured with a microtoise (SECA Bodymeter 206, Hamburg, Germany). All measurements were taken by trained research personnel. The body mass index (BMI) was calculated and categorised using World Health Organisation (WHO) BMI-for-age *z*-scores (5 to 19 years old) [34], while the BMI *z*-scores were calculated using WHO AnthroPlus software version 1.0.4 (WHO, Geneva, Switzerland) [35].

#### 2.2.3. Dietary Intakes Measurement and Assessment

Dietary intake data was collected from children using interviewer-administered 24-h recalls (one weekday and one weekend) assisted by food photographs, local household measures, and food packaging. The repeated diet recalls were based on a five pass method, adapted from the United States Department of Agriculture’s Automated Multiple Pass Method and the 24-h dietary recall procedures of Australia’s National Nutrition Survey 1995 [36,37]. The dietary data was supplemented with a food habits questionnaire (FHQ) adapted from the supplementary section of an Australian food frequency questionnaire with permission [38]. The FHQ had 13-items and aimed to provide information on the children’s usual food habit behaviours over a 6-month period. Information included the frequency of general food groups (fruits, vegetables, and dairy products), dietary habits (sweetened beverages, snacking, breakfast intake, eating out, and supplements), and sedentary behaviours (time spent watching television and playing video games). The FHQ was translated into the Malay language with local food terms verified by an independent linguistic department.

The individual mean daily nutrient intakes were analysed using Nutritionist Pro™ Diet Analysis (Axxya Systems, Washington, DC, USA), utilizing the Malaysian Nutrient Composition of Foods [39] and complemented by the Food Composition Guide Singapore [40]. Additional recipes and nutrient content or supplement information were entered into the database software. The proposed Black and Cole method (using 95% confidence limits of agreement between energy intake and total energy expenditure, measured by doubly labelled water) [41] was applied to identify energy misreporting, and dietary outliers were checked [42]. The basal metabolic rate (BMR) was estimated using the Malaysian-specific equations for children [43], while a physical activity level factor of 1.55 [44] was used to estimate total energy expenditure (TEE) [45]. Classification of misreporters was based on the ratio of reported energy intake to TEE, according to whether the individual’s ratio was within, below, or above the 95% confidence limits of the expected ratio of 1.0 (<0.76, under-reporter; 0.76–1.24, acceptable; >1.24, over-reporter). In view of the absence of national dietary intake data for children below 12 years of age at the point of designing the Family Diet Study, the total energy and nutrient intake values were compared with the age-relevant Recommended Nutrient Intakes (RNI) for Malaysia [46]. The RNI is the daily intake corresponding to the Recommended Daily Allowance, which meets the nutrient requirements of healthy individuals [46].

Foods obtained from the 24-h recalls were first divided into six major food groups and nine sub-groups. Mixed dishes were assigned a major food group based on primary ingredients, e.g., fried rice was assigned to ‘cereals/tubers/grains’. Nine food groups based on the Malaysian Food Pyramid [47] and the similarities of the items’ physical or preparation characteristics [20] were identified (Table S1): cereals; fruits/vegetables; meats; dairy; sugar-sweetened beverages; western fast food; snacks; sweets; and oils. A tenth group, ‘mixed food’, was developed for foods with no single food group accounting for at least 60% of their weight. The number of servings for the six main food groups was calculated by aggregating the total amounts for each food group and dividing by the standard serving size from the Malaysian Dietary Guidelines (MDG) [47]. The number of daily servings consumed was compared to MDG recommendations [47].

#### 2.2.4. Statistical Analysis

Analyses were carried out using STATA (Version 11.2, StataCorp, College Station, TX, USA). Descriptive statistics were applied to socio-demographic, anthropometry, and dietary intake data. Normality checking found data distributions to be either normally distributed (anthropometry and macronutrients) or skewed to the right (micronutrients and food groups). Parametric and non-parametric tests were used respectively for comparisons by gender, national recommendations, and body weight categories. Dietary intakes were tested for associations with the BMI *z*-scores for the sample and for plausible energy reporters using chi-squared tests and Pearson/Spearman correlation tests based on normality. Dietary variables with the highest adjusted coefficient of determination, *R*^2^, and value *p* < 0.05 from the univariate regressions were retained for subsequent multivariate linear regression model building using a forward stepwise approach. Age and gender were included in the model to control for potential confounders, as these characteristics were found to be associated with the study participants’ dietary variables and BMI *z*-scores.

## 3. Results

### 3.1. Study Participants

A total of 1372 invitations were sent by researchers working across five schools. Of the 793 invitations returned, 53% (*n* = 420) provided parental consent and child assent. After eligibility screening, 105 families were excluded for not meeting the inclusion criteria (predominantly due to inability to attend the required assessment session, *n* = 98) and 315 families were enrolled in the study. Of these, 236 participants completed all measures and were eligible for analysis.

#### 3.1.1. Socio-Demographic

Socio-demographic data are shown in Table 1. Slightly more than half of the children were female (52.5%). The mean (95% Confidence Interval (CI)) child age was 9.9 (9.8–10.0) years. A large proportion of parents reported that they worked full-time (76.9% fathers and 49.4% mothers), while a third of mothers were housewives. 90% of parents had attained at least secondary level education, and most had at least five family members living together (79.3%). Two-thirds of the families reported a monthly income above MYR2500 (estimated USD705), indicative of medium to high socio-economic status within the context of this developing country.

#### 3.1.2. Anthropometry

The mean (95% CI) body weight and height were 32.3 (31.0–34.6) kg and 133.2 (132.2–134.2) cm, respectively (Table 1). Using the WHO 2007 BMI-for-age, [34] 10.2% of participants were in the thinness/severe thinness category, 60.2% of the children were classified as normal weight, and one-third were either overweight (13.1%) or obese (16.5%). Similar distributions were observed for both genders (*p* > 0.05). The results on parental weight status have been published elsewhere [48]. Briefly, the mean (95% CI) BMI for fathers was 25.9 (25.0, 26.8) kg∙m^−2^ and mothers 27.1 (26.4, 27.8) kg∙m^−2^, with positive associations found between the child’s BMI and parental BMI [48].

There were no gender differences in the duration of television watching, using the computer, or playing video games (Table 1). The majority of the sample watched television for 3 h/day or less (72.1%) and played computer/video games twice a week or less (69.1%).

#### 3.1.3. Dietary Intake

Approximately one-third of the children were classified as energy misreporters, with 141 reporting plausible intakes [41]. The plausibility of energy intake was determined using the Black and Cole method [41]. Significantly more overweight and obese children than underweight children were identified as under-reporters (57.4% vs. 5.6% respectively). There were significantly more over-reporters amongst the underweight children than other weight categories (22.0% vs. 4.9%) (*x*^2^ = 35.46, *p* < 0.001) (Table S2). Home was the main venue for breakfast (77.8%), and 6.5% of children reported skipping breakfast, with no significant difference observed by gender or body weight status. The mean (95% CI) energy, carbohydrate, protein, and fat intake per day was 1698 (1637–1759) kcal, 229.0 (220.0–238.0) g, 65.0 (62.0–67.0) g, and 58.0 (56.0–61.0) g, respectively. Girls had significantly higher intakes of Vitamin C and calcium compared to boys (36.1 mg vs. 24.8 mg, *p* = 0.007; 411.7 mg vs. 349.9 mg, *p* = 0.007, respectively). The majority of the children reported not consuming fruits, legumes, or dairy every day. Girls reported higher intakes of condiments (*p* = 0.03), mixed food (*p* = 0.015), and pastries and dessert (*p* < 0.001) than boys (Table S3). Table 2 summarizes child dietary intakes in comparison with two age-specific reference standards, RNI [46] and MDG [47]. 

As can be seen in Table 2, the mean energy intake for girls under 10 years of age was significantly higher than the RNI (1803 kcal vs. 1590 kcal, *p* = 0.0041). For children 10–12 years of age, only 7.4% of boys and 18.6% of girls met the RNI of 2180 kcal and 1990 kcal, respectively. The RNI for protein was exceeded by the majority of the children of both age groups and genders, with intakes ranging from 132% up to 216%. Within each age sub-group, a large proportion of children did not meet the RNI for thiamin (75.4%–96.3%), niacin (75.4%–94.9%), and calcium (83.1%–100%). While the recommended daily number of servings for the ‘meat/poultry/fish’ group was met, other food groups were well below the national recommendations (*p* < 0.001; Table 2).

#### 3.1.4. Dietary Intakes by Body Weight Status

As shown in Table 3 (all children), children with obesity reported significantly lower energy intakes compared to children in the other weight categories, after energy intake was adjusted for body weight (kcal/kg) (F = 36.21, *p* < 0.001). Children with obesity reported consuming more ‘cereals’ and fewer ‘sugar sweetened beverages’ (grams) than did children in the healthy weight range (F = 2.74, *p* = 0.029; F = 11.04, *p* = 0.012, respectively). There were no other significant differences between dietary intakes and BMI categories (Table 3). When examining BMI *z*-scores (Table 4), there were three positive relationships with the intake of Vitamin A (*r* = 0.20, *p* = 0.002), iron (*r* = 0.13, *p* = 0.044), and the ‘cereals’ groups (*r* = 0.14, *p* = 0.034). The children’s reported energy intake, adjusted for body weight, was moderately strong and inversely correlated with their BMI *z*-scores (*r* = −0.59, *p* < 0.001).

In the sub-group analysis of plausible energy reporters (*n* = 141), significant differences were found between BMI categories and dietary intakes for energy, all macronutrients, and some micronutrients (Table 3). The BMI *z*-score was significantly associated with energy intake (*r* = 0.53), macronutrients (carbohydrate (*r* = 0.39), protein (*r* = 0.34) and fat (*r* = 0.40)], micronutrients (thiamin (*r* = 0.25), riboflavin (*r* = 0.30), niacin (*r* = 0.36), vitamin A (*r* = 0.31), iron (*r* = 0.37), and calcium (*r* = 0.27)). The BMI *z*-score was also associated with intakes of the ‘cereals’ (*r* = 0.23), and ‘meats’ (*r* = 0.18) groups (Table 4).

Univariate regression coefficients and their 95% confidence intervals are shown in Table 4. Multiple regression analysis indicated that total energy intake (β = 0.0029, *p* < 0.001), iron (β = 0.0261, *p* = 0.006), energy adjusted for body weight (β = −0.0988, *p* < 0.001), and age (β = −0.6598, *p* < 0.001) were significant predictors of a child’s BMI *z*-score. In the final model, these dietary intakes (total energy, iron, and energy adjusted for body weight) explained 86.0% of the variation in BMI *z*-scores amongst children with plausible energy reporting.

## 4. Discussion

This study aimed to comprehensively describe the dietary intake of urban Malay children aged between 8 and 12 years by body weight status. The results extend previous research [8,49,50] by describing the dietary intakes of the children in terms of food groups, macro- and micronutrient profiles (both vitamins and minerals), and the associations with body weight. The main findings were that majority of the children had dietary intakes below the national recommendations for nutrients and serving sizes, despite a prevalence of 30% overweightness and obesity in this population. This was largely affected by energy misreporting, explaining the contradictory results when only plausible energy reporters were included. The multivariable analyses support the hypothesis of positive associations between body weight status and energy intake.

About 10% of the children were classified as underweight and 30% were either overweight or obese, consistent with other paediatric studies in Malaysia [8,51] and regionally in Asia [1]. These findings of child body weight status highlighted the dual burden of under-nutrition and over-nutrition, a common problem observed in developing countries [17]. The nutrition transition has adversely influenced the behavior and social and cultural drivers within the obesogenic environment, which is likely to contribute to the onset and rapid growth of childhood obesity in these countries [6]. Given the findings of relatively higher socio-economic status amongst the majority of the participants in this study, the existence of child under-nutrition among the urban communities warrants further attention.

The proportion of children classified as underweight is higher than the 6.4% in the recent nationwide nutrition survey in 2013 [8] but closer to the 11.4% from the National Health and Morbidity Survey of 2006 [52]. The difference in rates was partly explained by the sampling protocol of these population surveys, in addition to the use of different comparisons to classify body weight status (WHO Child Growth Standards vs. the Centre of Disease Control Growth Charts) [34,53]. The key distinction between a growth reference and a growth standard is the development process and the application of growth charts [53]. Hence, it is essential to use the correct reference or standard based on the targeted population to improve the representativeness of the study findings. In this research, the WHO Child Growth Standards were applied due to the absence of national growth charts in Malaysia.

The prevalence of breakfast skipping in this sample was lower than that reported in similar age groups locally [27,29,50] and worldwide [54,55]. The lack of a relationship between breakfast skipping and BMI is at variance with the findings of studies that report a positive association between breakfast skipping and higher BMI among children and adolescents observed across different countries [27,50,54,55]. Suitable healthy breakfast food choices could be equally as important as the frequency of breakfast consumption. [54] It has been suggested that breakfast eaters had an overall nutritious diet and were more likely to meet recommended B vitamins, calcium, magnesium, and iron intakes [56]. Findings from the current study concurred with a local dietary study [29] indicating that the common Malay breakfast choices included fried rice, fried noodles, and nasi lemak (coconut milk steamed rice with condiments). These food choices are energy-dense, but low in micronutrients, which could adversely impact a child’s nutrient adequacy and diet quality.

Until recently, Malaysia has had no national nutrition and dietary intake data available for children aged below 12 years [8]. Consistent with other Malaysian dietary studies, we found that the majority of children 10–12 years of age did not meet the RNI for energy [8,49,57,58]. The majority of children did not meet key micronutrient recommendations (thiamin [8,57], niacin [8,57], and calcium [8,57,58]) nor the recommended number of servings for major food groups, except for ‘meats’ [49]. The lower than recommended energy and nutrient intakes reported in this sample are reflected in the median number of servings for the major food groups. Given the high prevalence of childhood obesity in the current study, the Malaysian RNI for these age groups could be too high, based on the FAO/WHO/UNU method of estimating energy requirements [46]. A review reported that most Malaysians maintained energy balance at relatively low energy intakes while leading a sedentary lifestyle [59]. Consumption of modified and refined grain products (e.g., fried rice, pastries and desserts) and processed meats (nuggets and sausages) contributed to a higher overall intake of protein and the ‘meats’ and ‘cereals’ groups, as well as increased total fat and decreased micronutrients intakes. Previous studies among children in United States have shown that energy-dense nutrient-poor foods were major contributors to total energy, fat, and carbohydrate intakes [60].

The large number of energy misreporters identified in the current study was consistent with another study of Malaysian children [49]. Excluding this group of energy reporters yielded contradicting results on the associations between dietary intakes and body weight status. The finding of a positive and strong relationship between Malay children’s energy intake and BMI *z*-scores is consistent with the hypothesis of how energy balance influences body weight [7,31]. The fact that the obese participants in this study reported the lowest energy intake adjusted for body weight compared to children in other weight categories corresponds well to other studies locally [28] and internationally [61,62]. We found a higher prevalence of under-reporters of energy intake amongst those with higher BMIs and a higher prevalence of over-reporters amongst those with lower BMIs, consistent with observations in Australian [63] and Japanese [64] children. While it is difficult to assess the independent effects of energy intake, such observations emphasize the influence of body weight and energy misreporting. Hence, the impact of both as potential confounders should be evaluated in dietary studies conducted in different populations. In addition, the increased awareness of childhood obesity in this developing country could influence the social desirability to under-report dietary intake. An alternative explanation might be that the children with obesity had lower energy requirements following metabolic and physiological adaptation [65], however in the absence of longitudinal data it is not possible to assess this.

Dairy intake, specifically milk intake, has been shown to be positively correlated with calcium intake in children. These dietary intakes are important for bone development; however, both were observed to be low in this study [66]. Poh et al. had previously reported a decrease in the prevalence of those meeting the RNI for calcium from infancy to primary-school aged children (17%–65%) [8]. The children in the current study also reported low intakes of non-dairy food sources of calcium such as green leafy vegetables and legumes. With no previous data on food consumption available for comparison, the significance of low calcium intake is unknown and its effect on the bone mineral status of Malaysian children remains unclear. While children consumed slightly more vegetables than fruit, their intakes were still well below the national recommendation of five servings of fruit and vegetables daily and much lower than the WHO population goals of ≥400 g per day [67]. The reported low intake of vegetables could be due to food neophobia [57] and a dislike of vegetables amongst young Malaysian children [68] and paediatric populations generally [69]. A lack of availability of fruit and vegetables at home may significantly influence the children’s actual consumption [49], emphasising the parental role in providing a healthy eating environment [70,71]. In addition, the household income could be another limiting factor to an adequate intake of fruit and vegetables, as suggested by findings from two local studies on children aged below 10 years [72,73], although income was relatively high by Malaysian standards in the current sample.

### Strengths and Limitations

The current study focuses on a relatively large sample size of the urban Malay population within the school setting to minimise variation in socio-demographic and food culture, shown to influence child dietary intakes [74]. The strength of this study included the involvement of trained personnel for data collection and the analysis of energy misreporting as a potential yet significant confounder of dietary intake. Despite the absence of direct energy expenditure measurements, the use of age-appropriate and local equations for BMR increases the accuracy of the estimation of TEE.

Limitations include the cross-sectional design of the study, which prevents examination of causal relationships between dietary factors and childhood obesity; the focus on the Malay population only, which limits the generalizability of the results beyond this ethnicity; and the high prevalence of energy misreporting as a source of error. Additional limitations include the self-reported measures by the children and the lack of precision when comparing to the broad RNI age group values rather than individually measured or estimated requirements. Direct measurement of physical activity of the children as an influence on overall energy balance was beyond the scope of this study. Apart from that, the lack of data on families who did not consent or did not complete the study introduces the possibility of selection bias.

## 5. Conclusions

Both over-nutrition and under-nutrition were observed in the current sample of urban Malay children, reflecting Malaysia’s nutrition transition. Despite a relatively high prevalence of overweightness and obesity, the majority of children had dietary intakes below recommended levels. Clear evidence for a positive and strong association between dietary intake and weight status was found after excluding energy misreporters. While the underlying key determinants of childhood obesity in developing countries are largely the same as in developed countries, these results have important implications for obesity-related research targeting Malay children. The current study emphasizes the need for further research and specific nutrition intervention programmes focussing on improved overall dietary patterns. Further validation work is warranted in the area of dietary assessment to minimise the impact of energy intake misreporting and to determine which cut-point method is most appropriate for identifying misreporters in this population.

## Figures and Tables

**Table 1:** Participant characteristics in The Family Diet Study.

	Total (*n* = 236)		Boys (*n* = 112)		Girls (*n* = 124)	
	*n* (%)					
**Family size**						
3	10 (4.2)		4 (4)		6 (5)	
4	39 (16.5)		20 (18)		19 (15)	
5	73 (30.9)		42 (3)		31 (25)	
6	53 (22.5)		20 (18)		33 (27)	
7 and above	61 (25.9)		26 (23)		35 (28)	
Family monthly total income *						
<1500	34 (14.4)		16 (14)		18 (15)	
1501–2500	59 (25.0)		23 (21)		36 (29)	
2501–3500	35 (14.8)		14 (13)		21 (17)	
3501–5000	43 (18.2)		22 (20)		21 (17)	
5001 and above	65 (27.5)		37 (33)		28 (23)	
Weekly pocket money given to child *						
<5.00	9 (3.8)		6 (5)		3 (2)	
5.01–9.99	16 (6.8)		5 (5)		11 (9)	
10.00–14.99	46 (19.5)		19 (17)		27 (22)	
15.00–19.99	61 (25.9)		29 (26)		32 (26)	
20.00 and above	104 (44.1)		53 (47)		51 (41)	
**Parental Employment Status**	**Fathers**	**Mothers**	**Fathers**	**Mothers**	**Fathers**	**Mothers**
Part-time	8 (3.6)	14 (6.0)	6 (6)	5 (5)	2 (2)	9 (7)
Full-time	173 (76.9)	115 (49.4)	78 (74)	59 (54)	95 (80)	56 (45)
Self-employed	42 (18.7)	32 (13.7)	21 (20)	14 (13)	21 (18)	18 (15)
Pensioner	2 (0.9)	0	1 (1)	0	1 (1)	0
Housewife	0	72 (30.9)	0	31 (28)	0	41 (33)
**Parental Highest Education Level**						
No formal education	5 (2.2)	4 (1.7)	3 (3)	2 (2)	2 (2)	2 (2)
Primary	19 (8.4)	19 (8.2)	7 (7)	9 (8)	12 (10)	10 (8)
Secondary	119 (52.7)	124 (53.5)	52 (49)	49 (45)	67 (56)	75 (61)
College/University	83 (36.7)	85 (36.6)	45 (42)	49 (45)	38 (32)	36 (29)
**Television Usage**						
≤1 h daily	54 (22.9)		22 (19.6)		32 (25.8)	
2 to 3 h daily	116 (49.2)		57 (50.9)		59 (47.6)	
4 to 5 h daily	44 (18.6)		22 (19.6)		22 (17.7)	
≥6 h daily	22 (9.3)		11 (9.8)		11 (8.9)	
**Computer or Video Game Usage**						
Never	49 (20.8)		15 (13.4)		34 (27.4)	
<Once a week	50 (21.2)		26 (23.2)		24 (19.4)	
1 to 2 weekly	64 (27.1)		31 (27.7)		33 (26.6)	
3 to 4 weekly	26 (11.0)		11 (9.8)		15 (12.1)	
5 to 6 weekly	16 (6.8)		9 (8.0)		7 (5.7)	
Daily	31 (13.1)		20 (17.9)		28 (30.4)	
	**Mean (CI)**					
**Age (year)**	9.9 (9.8–10.0)		9.8 (9.7–10.0)		9.9 (9.8–10.1)	
**Body Weight (kg)**	32.3 (31.0–34.6)		32.0 (30.2–33.8)		32.6 (30.6–34.6)	
**Height (cm)**	133.2 (132.2–134.2)		132.7 (131.3–134.2)		133.5 (132.2–134.9)	
**Body Mass Index (kg/m^2^)**	17.9 (17.4–18.5)		17.9 (17.2–18.6)		18.0 (17.1–18.9)	
**BMI *z*-score**	0.20 (–0.01–0.42)		0.34 (0.03–0.64)		0.08 (−0.22–0.39)	
**BMI classification**	***n* (%)**					
*z* < −3SD (Severe thinness)	3 (1.3)		1 (1)		2 (2)	
−3SD < *z* < −2SD (Thinness)	21 (8.9)		7 (6)		14 (11)	
−2 SD < *z* < +1SD (Normal weight)	142 (60.2)		70 (63)		72 (58)	
+1SD < *z* < +2SD (Overweight)	31 (13.1)		16 (14)		15 (12)	
*z* > +2 SD (Obesity)	39 (16.5)		18 (16)		21 (17)	

*n*, number of participants; * MYR, Malaysian Ringgit; SD, Standard Deviation; IQR, Inter-quartile Range; CI, Confidence Interval; BMI, body mass index.

**Table 2:** Dietary intakes of children compared with the Malaysian Recommended Nutrient Intake (RNI) and Malaysian Dietary Guidelines Recommended Serving Size (RSS).

Gender	Age	Nutrient	Mean (95% CI)	% RNI	<RNI, No. (%)	RNI	Food Groups	Mean (95% CI)	% RNI	<RSS, No. (%)	RSS
Boys (*n* = 112)	8–9.9 years (*n* = 58)	Energy (kcal)	1639 (1536–1742) *	92	39 (67) **	1780	Cereals/tubers/grains	4.0 (3.6–4.4) *	81	47 (81)	5
		Protein (g)	64 (58–70) *	200	6 (10)	32	Fruits	0.3 (0.2–0.4) *	16	57 (98)	2
		Thiamin (mg)	0.7 (0.6–0.8) *	75	46 (79)	0.9	Vegetables	0.4 (0.2–0.6) *	12	58 (100)	3
		Riboflavin (mg)	1.0 (0.9–1.1)	111	28 (48) **	0.9	Meat/poultry/fish	2.3 (1.9–2.7)	116	27 (47)	2
		Niacin (mg)	8 (7–9) *	68	46 (79)	12	Legumes	0.06 (0.02–0.10) *	37	58 (100)	1
		Vitamin C (mg)	39 (27–51)	111	38 (65) **	35	Milk and dairy products	0.2 (0.1–0.3) *	11	58 (100)	2
		Vitamin A (µg)	599 (522–676) *	120	25 (43)	500					
		Iron (mg)	14 (12–16) *	156	14 (24)	9					
		Calcium (mg)	423 (367–479) *	61	51 (88)	700					
	10–12 years (*n* = 54)	Energy (kcal)	1667 (1556–1778) *	77	50 (93) **	2180	Cereals/tubers/grains	4.0 (3.6–4.4) *	57	51 (94)	7
		Protein (g)	63 (57–69) *	141	12 (22)	45	Fruits	0.2 (0.1–0.3) *	10	54 (100)	2
		Thiamin (mg)	0.6 (0.5–0.7) *	50	52 (96)	1.2	Vegetables	0.5 (0.2–0.8) *	17	52 (96)	3
		Riboflavin (mg)	0.9 (0.8–1.0) *	66	49 (91) **	1.3	Meat/poultry/fish	2.3 (2.0–2.6)	90	32 (59)	2.5
		Niacin (mg)	8 (7–9) *	49	50 (93)	16	Legumes	0.20 (–0.002–0.4) *	50	51 (94)	1
		Vitamin C (mg)	44 (30–58) *	68	42 (78) **	65	Milk and dairy products	0.08 (0.02–0.13) *	4	54 (100)	2
		Vitamin A (µg)	612 (511–713)	102	34 (63)	600					
		Iron (mg)	15 (13–17)	99	34 (63)	15					
		Calcium (mg)	358 (306–410) *	36	54 (100)	1000					
Girls (*n* = 124)	8–9.9 years (*n* = 65)	Energy (kcal)	1803 (1660–1946) *	113	25 (39) **	1590	Cereals/tubers/grains	3.9 (3.6–4.3) *	78	51 (79)	5
		Protein (g)	69 (64–74) *	216	1 (2)	32	Fruits	0.4 (0.2–0.6) *	21	64 (99)	2
		Thiamin (mg)	0.7 (0.6–0.8) *	83	49 (75)	0.9	Vegetables	0.5 (0.3–0.7) *	16	63 (97)	3
		Riboflavin (mg)	1.1 (1.0–1.2) *	118	31 (48) **	0.9	Meat/poultry/fish	2.4 (2.1–2.7) *	120	27 (42)	2
		Niacin (mg)	9 (8–10)	78	49 (75)	12	Legumes	0.06 (0.01–0.10) *	98	63 (97)	0.5
		Vitamin C (mg)	59 (46–72) *	168	27 (42) **	35	Milk and dairy products	0.33 (0.19–0.47) *	17	63 (97)	2
		Vitamin A (µg)	663 (559–767) *	133	29 (45)	500					
		Iron (mg)	14 (12–16) *	158	14 (22)	9					
		Calcium (mg)	496 (424–568) *	71	54 (83)	700					
	10–12 years (*n* = 59)	Energy (kcal)	1669 (1540–1798) *	84	48 (81) **	1990	Cereals/tubers/grains	4.0 (3.6–4.4) *	60.0	58 (98)	6
		Protein (g)	61 (55–67) *	132	16 (27)	46	Fruits	0.3 (0.2–0.4) *	14.1	58 (98)	2
		Thiamin (mg)	0.6 (0.5–0.7) *	56	55 (93)	1.1	Vegetables	0.4 (0.2–0.6) *	14.8	59 (100)	3
		Riboflavin (mg)	0.9 (0.9–1.3) *	91	35 (59) **	1.0	Meat/poultry/fish	1.9 (1.6–2.2)	95.4	34 (58)	2
		Niacin (mg)	8 (7–9) *	48	56 (95)	16	Legumes	0.14 (0.03–0.25) *	44.5	58 (98)	1
		Vitamin C (mg)	66 (42–90)	101	43 (73) **	65	Milk and dairy products	0.21 (0.09–0.33) *	10.4	58 (98)	2
		Vitamin A (µg)	705 (480–930)	118	40 (68)	600					
		Iron (mg)	13 (11–15)	96	35 (59)	14					
		Calcium (mg)	454 (393–515) *	45	58 (98)	1000					

* Significant difference (*z*-test comparison with RNI/ RSS); ** Significant difference (Pearson Chi-squared test by gender for intake below RNI/RSS).

**Table 3:** Differences in dietary intakes by BMI category for all children and for plausible energy reporters.

	Total (*n* = 236)				Plausible Reporters (*n* = 141)			
Nutrients/Food Groups	BMI Category							
	Thinness (*n* = 24)	Normal (*n* = 142)	Overweight (*n* = 31)	Obese (*n* = 39)	Thinness (*n* = 12)	Normal (*n* = 93)	Overweight (*n* = 17)	Obese (*n* = 19)
	Mean (SD)							
**Energy (kcal) ^a^**	1695 (532)	1693 (479)	1623 (434)	1778 (488)	1502 (273) ***	1613 (219) ***	1873 (262) ***	2026 (298) ***
**Carbohydrate (g) ^a^**	232.4 (65.1)	226 (71.4)	224.9 (58.3)	240.6 (69.1)	216.3 (45.8) **	213.7 (38.9) **	251.8 (43.3) **	274.2 (48.5) **
**Protein (g) ^a^**	63.6 (25.9)	64.9 (22.4)	60.5 (19.9)	66.4 (25.1)	54.0 (14.7) *	63.2 (18.0) *	71.2 (16.6)	76.8 (19.3) *
**Fat (g) ^a^**	56.2 (23.1)	59.1 (20.6)	53.6 (20.7)	60.8 (21.0)	46.7 (11.8) **	56.3 (12.5) **	64.1 (13.7) **	68.7 (16.0) **
**Energy/ body weight (kcal/kg) ^a^**	78.1 (26.5) ***	62.0 (19.8) **	42.3 (12.0) ***	36.5 (10.2) ***	69.2 (9.2) ***	59.0 (10.4) ***	49.7 (6.5) ***	41.9 (7.8) ***
**Percent energy from carbohydrate (%) ^a^**	55.7 (6.1)	53.3 (6.4)	55.9 (6.8)	54.4 (6.3)	57.9 (7.0) *	52.9 (5.7) *	54.0 (4.8)	54.2 (5.8)
**Percent energy from protein (%)**	14.7 (2.7)	15.4 (3.7)	15.0 (3.0)	14.9 (3.4)	14.3 (3.1)	15.7 (3.6)	15.3 (3.0)	15.3 (3.9)
**Percent energy from fat (%)**	29.6 (4.8)	31.3 (5.5)	29.1 (6.0)	30.6 (4.6)	27.8 (4.8)	31.4 (5.2)	30.8 (4.6)	30.4 (4.3)
**Cereals (g) ^a^**	413.1 (140.0)	397.7 (161.2) *	405.7 (143.4)	482.2 (199.7) *	412.8 (151.6)	393.0 (152.9) *	449.9 (146.4)	520.9 (245.3) *
	**Median (IQR)**							
**Thiamin (mg) ^b^**	0.6 (0.3)	0.6 (0.4)	0.6 (0.3)	0.6 (0.3)	0.5 (0.2)	0.6 (0.4)	0.7 (0.3)	0.8 (0.3)
**Riboflavin (mg) ^b^**	0.9 (0.6)	0.8 (0.5)	0.9 (0.6)	1.0 (0.8)	0.8 (0.4) **	0.8 (0.4) **	1.1 (0.4) **	1.3 (0.6) **
**Niacin (mg) ^b^**	7.9 (5.2)	7.3 (6.2)	7.8 (7.7)	7.0 (4.3)	6.0 (2.8) **	7.3 (5.4)	11.3 (5.1) **	9.1 (4.0)
**Vitamin C (mg) ^b^**	45.6 (43.1)	27.5 (52.2)	35.1 (53.5)	26.3 (58.9)	39.0 (31.9)	27.6 (47.5)	53.4 (59.7)	38.1 (78.0)
**Vitamin A (µg) ^b^**	440.3 (349.7)	513.9 (408.9)	508.7 (317.8)	684.3 (516.5)	424.0 (230.0) **	498.3 (342.3) **	533.6 (280.4)	847.3 (613.1) **
**Iron (mg) ^b^**	11.9 (9.4)	11.9 (8.4)	14.2 (7.5)	12.9 (8.7)	10.2 (5.2)	11.7 (7.0) **	15.5 (6.2)	15.7 (8.2) **
**Calcium (mg) ^b^**	346.9 (200.8)	374.7 (304.0)	383.8 (266.6)	376.3 (209.0)	336.6 (138.7)	365.9 (246.3)	452.8 (241.0)	455.8 (306.8)
**Fruits/vegetables (g) ^b^**	54.5 (138.8)	28.5 (97.0)	32.0 (107.5)	66.0 (143.5)	27.8 (91.8)	29.5 (92.0)	35.5 (111.0)	16.0 (209.5)
**Meats (g) ^b^**	113.0 (65.0)	113.5 (119.5)	168.0 (114.0)	115.5 (199.0)	118.3 (47.3)	115.5 (115.0)	176.0 (113.5)	219.0 (215.0)
**Dairy (g) ^b^**	0 (0)	0 (75.0)	0 (10.5)	0 (80.0)	0 (0)	0 (15.8)	0 (12.3)	0 (100.0)
**Sugar sweetened beverages (g) ^b^**	143.0 (155.0)	140.0 (250.0) *	193.0 (250.0) *	48.5 (141.0) *	122.8 (214.0)	133.0 (247.0)	237.5 (220.5)	48.5 (317.0)
**Sweets (g) ^b^**	47.8 (115.3)	62.5 (68.3)	63.0 (83.5)	73.1 (96.2)	40.0 (89.8)	61.5 (60.7)	70.0 (79.5)	77.0 (82.0)
**Oils (g) ^b^**	28.5 (38.3)	20.5 (29.0)	30.0 (35.0)	26.0 (36.5)	22.3 (38.0)	23.0 (26.0)	33.5 (36.0)	27.0 (43.0)
**Snack (g) ^b^**	0.5 (30.5)	4.9 (27.1)	0 (15.3)	0 (21)	1.5 (30.5)	4.9 (21.0)	0 (15.3)	0 (21.0)
**Western fast food (g) ^b^**	0 (0)	0 (24.0)	0 (0)	0 (0)	0 (0)	0 (31.5)	0 (0)	0 (0)
**Mixed food (g) ^b^**	0 (0)	0 (0)	0 (0)	0 (0)	0 (0)	0 (0)	0 (0)	0 (0)

BMI: Body Mass Index; SD: Standard Deviation; IQR: Inter-quartile Range; ^a^ ANOVA F-test by body weight status, ^b^ Kruskal-Wallis by body weight status; Significant difference by BMI category (* *p* < 0.05, ** *p* < 0.01, *** *p* < 0.001).

**Table 4:** Dietary intakes and BMI *z*-scores for all children and for plausible energy reporters.

Nutrients/Food Groups	Total Children (*n* = 236)	Plausible Reports (*n* = 141)	
	BMI *z*-Score		
	CC, *r*	CC, *r*	Regression Coefficients ^e^
Energy (kcal)	0.04	0.53 ^c,^***	0.003 (0.002,0.004) ***
Carbohydrate (g)	0.01	0.39 ^c,^***	0.014 (0.008,0.019) ***
Protein (g)	0.03	0.34 ^c,^***	0.029 (0.016, 0.043) ***
Fat (g)	−0.04	0.40 ^c,^***	0.046 (0.028, 0.064) ***
Energy/body weight (kcal/kg)	−0.59 ^c^^,^***	−0.68 ^c,^***	−0.092 (−0.109, −0.075) ***
Percent energy from carbohydrate (%)	0.02	−0.08	−0.022 (−0.068, 0.025)
Percent energy from protein (%)	0.04	0.05	0.021 (−0.056, 0.098)
Percent energy from fat (%)	0.05	0.06	0.018 (−0.035, 0.072)
Thiamin (mg)	−0.02	0.25 ^d,^**	1.240 (0.357, 2.124) *
Riboflavin (mg)	0.03	0.30 ^d,^***	1.400 (0.736, 2.062) ***
Niacin (mg)	0.009	0.36 ^d,^***	0.130 (0.065, 0.194) ***
Vitamin C (mg)	−0.06	0.06	−0.0002 (−0.0041, 0.0037)
Vitamin A (µg)	0.20 ^d,^**	0.31 ^d,^***	0.0007 (0.0001, 0.0013) *
Iron (mg)	0.13 ^d,^*	0.37 ^d,^***	0.093 (0.053, 0.134) ***
Calcium (mg)	0.06	0.27 ^d,^**	0.002 (0.001, 0.004) **
Cereals (g)	0.14 ^c,^*	0.23 ^c,^**	0.002 (0.001, 0.004) **
Fruits/vegetables (g)	0.02	0.005	0.0006 (−0.0021, 0.0033)
Meats (g)	0.07	0.18 ^d,^*	0.004 (0.001, 0.007) **
Dairy (g)	−0.006	0.06	0.003 (−0.0004, 0.0056) *
Sugar-sweetened beverages (g)	−0.10	−0.004	−0.0003 (−0.0016, 0.0010)
Snacks (g)	−0.13 ^d,^*	−0.14	−0.004 (−0.014, 0.006)
Western fast food (g)	−0.01	0.005	0.002 (−0.003, 0.008)
Sweets (g)	−0.01	0.11	0.002 (−0.002, 0.006)
Oils (g)	0.08	0.11	0.006 (−0.004, 0.015)

CC, correlation coefficient; ^c^ Pearson correlation coefficient; ^d^ Spearman correlation coefficient; ^e^ Univariate coefficients and 95% confidence intervals; Significant (* *p* < 0.05, ** *p* < 0.01, *** *p* < 0.001).

## Supplementary Materials

The following are available online at www.mdpi.com/1999-4915/4/01/05/s1: Table S1: Food grouping scheme; Table S2: Classification of energy misreporting for all children sorted by gender and BMI category; Table S3: Characteristics of children’s dietary intakes (energy, macronutrients, micronutrients, and food groups).
