# Supplementary material for: Body Weight Status and Dietary Intakes of Urban Malay Primary School Children: Evidence from the Family Diet Study
Source: Children (Basel). 2017 Jan 20;4(1):5. doi: 10.3390/children4010005 (PMC5296666; doi:10.3390/children4010005)
Supplement: Supplementary file 1 [file children-04-00005-s001.pdf]

Supplementary Materials: Body Weight Status and Dietary Intakes of Urban Malay Primary School Children: Evidence from the Family Diet Study

Wai Yew Yang, Tracy Burrows, Lesley MacDonald-Wicks, Lauren T. Williams, Clare E. Collins, Winnie Siew Swee Chee, and Kim Colyvas

Table S1: Food grouping scheme.

	Major Food Groups	Sub Food Groups
STEP 1	Cereals/tubers/grains<br>Fruits<br>Vegetables<br>Meat/poultry/fish<br>Legumes<br>Milk and dairy products	Sugar-sweetened beverages<br>Western fast food<br>Pastries and dessert (Local & Western)<br>Snacks<br>Spreads<br>Sugar<br>Oils<br>Condiments<br>Mixed food
	**Combined Food Groups**	
STEP 2	Cereals (Cereals/tubers/grains)<br>Fruits/vegetables<br>Meats (Meat/poultry/fish/legumes)<br>Dairy (Milk/dairy products)<br>Sugar-sweetened beverages<br>Western fast food<br>Snacks<br>Sweets (Pastries/desserts/sugar)<br>Oils (spreads/oils/condiments) Mixed food	

Table S2. Classification of energy misreporting for all children sorted by gender and BMI category.

Classification of Energy Mis-reporting [a]	All Children (*n* = 236)	Gender		BMI Category			
		Boys (*n* = 112)	Girls (n = 124)	Thinness (*n* = 24)	Normal (*n* = 124)	Overweight (*n* = 31)	Obese (*n* = 39)
Under-reporter	54 (22.9)	31 (28) *	23 (19)	3 (13) *	20 (14)	13 (42) *	18 (46) *
Plausible reporter	141 (59.8)	68 (61)	73 (59)	12 (50)	93 (66)	17 (55)	19 (49)
Over-reporter	41 (17.4)	13 (12) *	28 (23)	9 (37) *	29 (20)	1 (3)	2 (5) *

BMI, Body Mass Index; [a], Black and Cole cut-point; *, Significant difference (Pearson Chi-squared test between gendesr; by body weight status).

Table S3. Characteristics of children's dietary intakes (energy, macronutrients, micronutrients, and food groups).

Energy and Macronutrients	Min	Average Daily Intake (*n* = 236) Mean (95% CI)			Max
Energy (kcal/d)	588	1698 (1637-1759)			3795
Carbohydrate (g/d)	88.1	229.1 (220.3-237.9)			536.8
Protein (g/d)	16.5	64.5 (61.5-67.4)			151.9
Fat (g/d)	15.2	58.4 (55.7-61.0)			142.1
Energy/ body weight (kcal/kg)	18.8	56.8 (54.0-59.7)			111.5
**Micronutrients**	**Min**	**25% Percentile**	**Median**	**75% Percentile**	**Max**
Thiamin (mg/day)	0.1	0.4	0.6	0.8	3.6
Riboflavin (mg/day)	0.1	0.6	0.9	1.2	3.8
Niacin (mg/day)	0.3	4.9	7.4	11.2	40.5
Vitamin C (mg/day) *	0	14.6	31.5	66.9	477.9
Vitamin A (μg/day)	68.7	383.5	521.9	800.9	5665.6
Iron (mg/day)	3.8	9.5	12.3	17.2	43.1
Calcium (mg/day) *	76.7	270.0	374.4	559.4	1715.5
Main Food Groups Cereals/tubers/grains g/day	0	294.3	397.5	532.5	941.5
Servings/day	0	3.0	3.7	4.7	7.8
Meat/poultry/fish (g)					
g/day	0	59.0	108.8	171.0	554.5
Servings/day	0	1.3	2.1	2.9	7.9
Vegetables (g) g/day	0	0	8.8	40.0	366.0
Servings/day	0	0	0.1	0.5	4.9
Fruits (g)					
g/day	0	0	0	55.0	300.0
Servings/day	0	0	0	0.4	3.6
Milk and dairy products (g)					
g/day	0	0	0	65.0	662.5
Servings/day	0	0	0	0.2	2.9
Legumes (g) g/day	0	0	0	0	300.0
Servings/day	0	0	0	0	4.6
Sub Food Groups Sugar-sweetened beverages (g/day)	0	18.0	134.0	274.0	1243.0
Sugar (g/day)	0	10.0	22.8	39.0	174.5
Condiments(g/day) *	0	2.5	10.0	19.4	112.5
Pastries and dessert (Local & Western) (g/day) *	0	0	30.0	81.5	778.5
Oils (g/day)	0	0	6.8	20.0	198.0
Snacks (g/day)	0	0	3.8	25.8	175.0
Western fast food (g/day)	0	0	0	0	681.9
Mixed food (g/day) *	0	0	0	0	515.0
Spreads (g/day)	0	0	0	0	40.0

*, Significant differences (Mann-Whitney test between gender).

© 2017 by the authors. Submitted for possible open access publication under the terms and conditions of the Creative Commons Attribution (CC-BY) license (http://creativecommons.org/licenses/by/4.0/).
